# Poziotinib Inhibits the Efflux Activity of the ABCB1 and ABCG2 Transporters and the Expression of the ABCG2 Transporter Protein in Multidrug Resistant Colon Cancer Cells

**DOI:** 10.3390/cancers12113249

**Published:** 2020-11-04

**Authors:** Yongchao Zhang, Zhuo-Xun Wu, Yuqi Yang, Jing-Quan Wang, Jun Li, Zoey Sun, Qiu-Xu Teng, Charles R. Ashby, Dong-Hua Yang

**Affiliations:** 1 Department of General Surgery, The Affiliated Cancer Hospital of Zhengzhou University/Henan Cancer Hospital, Zhengzhou 450003, China; 2 Department of Pharmaceutical Sciences, College of Pharmacy and Health Sciences, St. John’s University, Queens, NY 11439, USA; zhuoxun.wu17@my.stjohns.edu (Z.-X.W.); yuqi.yang17@my.stjohns.edu (Y.Y.); jingquan.wang16@my.stjohns.edu (J.-Q.W.); sunz2@bxscience.edu (Z.S.); qiuxu.teng15@my.stjohns.edu (Q.-X.T.); cnsratdoc@optonline.net (C.R.A.J.); 3 Department of Otolaryngology-Head and Neck Surgery, Zhongnan Hospital of Wuhan University, Wuhan 430071, China; jun-li@whu.edu.cn

**Keywords:** multidrug resistance, ABCB1, ABCG2, poziotinib, EGFR inhibitor, colorectal cancer

## Abstract

**Simple Summary:**

Globally, colorectal cancer (CRC) is a leading cause of cancer deaths and chemotherapy, in combination with radiotherapy when appropriate, is used to treat the majority of CRC patients. However, the acquisition or development of drug resistance can decrease, or even abolish, the efficacy of chemotherapy. ATP-binding cassette (ABC) transporters, particularly, the ABCB1 and ABCG2 transporter, are mediators of multidrug resistance (MDR) in certain types of cancer cells. The aim of our in vitro study was to determine if poziotinib can overcome MDR to certain chemotherapeutic drugs in colon cancer cells. Our results indicated that in MDR CRC cell lines, poziotinib inhibits the transport function of the ABCB1 and ABCG2 transporters, increasing the intracellular accumulation of certain anticancer drugs, and thus, their efficacy. Furthermore, poziotinib decreased the expression of the ABCG2 protein. Therefore, if our results can be translated to humans, they suggest that using poziotinib in combination with certain anticancer drugs may be of therapeutic benefit in colorectal cancer patients.

**Abstract:**

Colorectal cancer (CRC) is a leading cause of cancer deaths in the United States. Currently, chemotherapy is a first-line treatment for CRC. However, one major drawback of chemotherapy is the emergence of multidrug resistance (MDR). It has been well-established that the overexpression of the ABCB1 and/or ABCG2 transporters can produce MDR in cancer cells. In this study, we report that in vitro, poziotinib can antagonize both ABCB1- and ABCG2-mediated MDR at 0.1–0.6 μM in the human colon cancer cell lines, SW620/Ad300 and S1-M1-80. Mechanistic studies indicated that poziotinib increases the intracellular accumulation of the ABCB1 transporter substrates, paclitaxel and doxorubicin, and the ABCG2 transporter substrates, mitoxantrone and SN-38, by inhibiting their substrate efflux function. Accumulation assay results suggested that poziotinib binds reversibly to the ABCG2 and ABCB1 transporter. Furthermore, western blot experiments indicated that poziotinib, at 0.6 μM, significantly downregulates the expression of the ABCG2 but not the ABCB1 transporter protein, suggesting that the ABCG2 reversal effect produced by poziotinib is due to transporter downregulation and inhibition of substrate efflux. Poziotinib concentration-dependently stimulated the ATPase activity of both ABCB1 and ABCG2, with EC_50_ values of 0.02 μM and 0.21 μM, respectively, suggesting that it interacts with the drug-substrate binding site. Molecular docking analysis indicated that poziotinib binds to the ABCB1 (−6.6 kcal/mol) and ABCG2 (−10.1 kcal/mol) drug-substrate binding site. In summary, our novel results show that poziotinib interacts with the ABCB1 and ABCG2 transporter, suggesting that poziotinib may increase the efficacy of certain chemotherapeutic drugs used in treating MDR CRC.

## 1. Introduction

In recent decades, colorectal cancer (CRC) has become one of the most common malignancies worldwide [1]. The mainstream therapies for patients with CRC includes irinotecan, 5-fluorouracil and oxaliplatin, as well as the target monoclonal antibody drugs, cetuximab, and trastuzumab [2]. One major drawback of chemotherapy is the emergence of multidrug resistance (MDR) in cancer cells [3]. MDR occurs when cancer cells, after exposure to a specific chemotherapeutic drug, become resistant to a broad array of anticancer drugs with unrelated structures and mechanism of actions [4]. There are numerous mechanisms that mediate MDR, including, but not limited to, inhibition of cell death pathways, overexpression of efflux pumps (notably, ATP-binding cassette (ABC) transporters), evasion of the immune response, mutations in cellular drug targets and sequestration of drugs in lysosomes [5]. Numerous studies indicate that one of the main mechanisms that mediates MDR in cancer cells is the overexpression of certain ABC transporters [6,7,8]. Indeed, the ABCG2 and ABCB1 transporters play a major role in MDR-related cancers [9]. These transporters are primarily expressed on the membrane of tissues and organs, including the intestines, blood-brain barrier, placenta and kidney, to mediate the efflux of endogenous and exogenous toxins, including certain anticancer drugs [10,11]. The anticancer drugs paclitaxel, doxorubicin and vincristine are substrates for the ABCB1 transporter [12], whereas mitoxantrone, topotecan and irinotecan are substrates for the ABCG2 transporter [13]. It has been reported that in CRC biopsy samples, there was a 10-fold increase in the ABCG2 transporter expression level after irinotecan treatment [14]. There are also data suggesting that the ABCG2 transporter is a potential biomarker for CRC [15,16]. Similarly, high ABCB1 expression levels were correlated with the developments of intrinsic drug resistance in colon cancer cell lines to daunorubicin and doxorubicin [17,18]. Furthermore, numerous in vitro and in vivo studies indicate that the inhibition of the efflux function and/or expression of the ABCB1 or ABCG2 transporter can significantly increase the efficacy of certain anticancer drugs in various types of cancers that overexpress these ABC transporters [19]. Therefore, it may be hypothesized that compounds with reversal MDR efficacy for both the ABCG2 and ABCB1 transporters could produce additional therapeutic benefits in CRC patients receiving chemotherapy anticancer drugs that are substrates for these transporters.

Over the last two decades, specific tyrosine kinase inhibitors (TKIs) have been reported to reverse MDR mediate by ABCG2 and ABCB1 transporters. For example, M3814 [20], rociletinib [21], TAE684 [22] and venetoclax [23] significantly attenuate or abolish ABCG2-mediated MDR, whereas tepotinib [24] and erdafitinib [25] overcome MDR mediated by the overexpression of the ABCB1 transporter. ABC transporters can also confer resistance to the TKIs, such as pevonedistat [26], PF-4989216 [27] and tivantinib [28], thereby attenuating their efficacy in cancer cells. One potential strategy is to combine TKIs that have MDR reversal efficacy with anticancer drugs to overcome MDR mediated by the ABCB1 or ABCG2 transporter. Past studies have suggested that several TKIs including glesatinib [29], AG1478 [30] and osimertinib [31] can reverse MDR in certain types of cancer cells. Therefore, we screened a panel of TKIs that are currently being evaluated in phase 2 or 3 clinical trials using the MTT assay to determine their MDR reversal efficacy. Poziotinib (HM781-36B) is a novel, irreversible EGFR/pan-HER and a member of the TEC family of nonreceptor/cytoplasmic TKI that is currently been evaluated for efficacy in the treatment of gastric cancer (NCT01746771), breast cancer (NCT02544997), non-small-cell lung cancer (NCT03066206, NCT03318939) and CRC (NCT04172597) [32,33,34,35]. The antitumor efficacy of poziotinib has been demonstrated in several cancer cell models, including gastric cancer, breast cancer and CRC [36,37,38]. Poziotinib is significantly more potent than other EGFR inhibitors, such as BIBW-2992, gefitinib and lapatinib, in inhibiting the proliferation of gastric cancer cells that overexpress the HER2 protein [37]. Furthermore, the EGFR tyrosine kinases are over-expressed in colorectal, lung and breast cancers [39,40]. It has been suggested that poziotinib, in combination with anticancer drugs such as 5-FUand oxaliplatin, may be efficacious in EGFR-overexpressing CRC cells [36].

In the present in vitro study, we report that poziotinib can reverse MDR in colon cancer cells by antagonizing the efflux function of the ABCB1 and ABCG2 transporters, as well as decreasing the expression of the ABCG2 transporter protein.

## 2. Results

### 2.1. The Cytotoxicity of Poziotinib in Parental and MDR Colon Cancer Cell Lines

**Figure 1:**
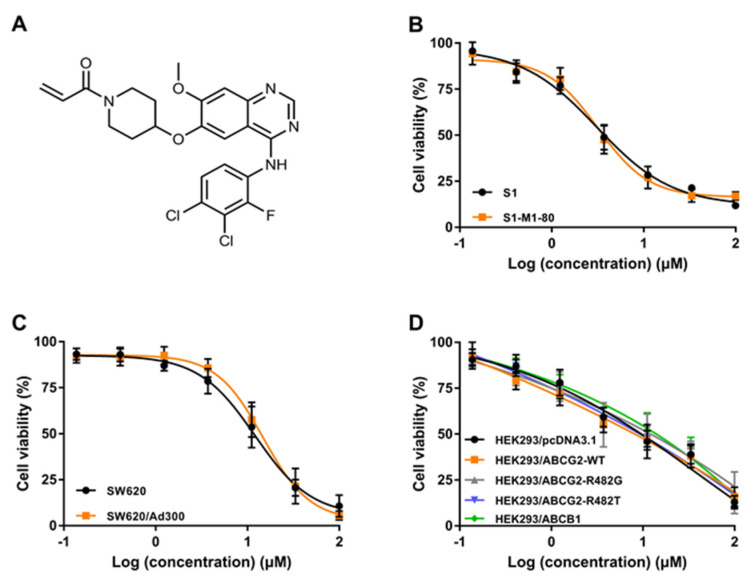
The cytotoxicity of poziotinib in parental and drug-resistant cell lines. (**A**) The chemical structure of poziotinib; cell viability curves for (**B**) S1 and S1-M1-80 colon cancer cells; (**C**) SW620 and SW620/Ad300 colon cancer cells and (**D**) the transfected HEK293/pcDNA3.1, HEK293/ABCB1, HEK293/ABCG2-WT, HEK293/ABCG2-R482G and HEK293/ABCG2-R482T cells. Data are expressed as mean ±SD based on data from three independent experiments.

The cytotoxicity of poziotinib was first determined in all cell lines used in the present study. To determine whether poziotinib reverses ABC transporter-mediated MDR, the colon cancer cell line, S1 and its mitoxantrone-selected, ABCG2-overexpressing subline, S1-M1-80 cells, SW620 and its doxorubicin-selected, ABCB1-overexpressing subline SW620/Ad300 cells, were used in this study. In addition, we used the *ABCG2* gene-transfected HEK293/ABCG2 and *ABCB1* gene-transfected HEK293/ABCB1 cells. This approach is important as these cells will be resistant to the anticancer drugs only as a result of their overexpression of these transporters and thus poziotinib’s reversal efficacy should be due solely to it effect on the ABCB1 and/or ABCG2 transporters. As shown in Figure 1, the cytotoxicity of poziotinib was similar in each pair of cell lines and no significant difference was observed in the non-toxic concentration of poziotinib between the cell lines. Therefore, based on these results, the non-toxic concentrations (0.1–0.6 μM) of poziotinib were chosen to minimize cytotoxicity in the poziotinib-anticancer drug combination experiments. 

### 2.2. Poziotinib Increases the Anticancer Efficacy of Substrate Chemotherapeutic Drugs in Colon Cancer Cells Overexpressing ABCG2 and ABCB1 Trasnporters

In these experiments, we determined the reversal effect of poziotinib on the efficacy of specific anticancer drugs in colon cancer cells overexpressing the ABCG2 or ABCB1 transporters and in HEK293 cells transfected with the *ABCG2* or *ABCB1* gene. In addition, we also determined the effect of Ko143 and verapamil, which are inhibitors of the ABCG2 and ABCB1 transporters, respectively, in the same cell lines, as positive controls. As shown in Table 1, the S1-M1-80 cells were markedly resistant to mitoxantrone (RF = 125.75) and SN-38 (RF = 97.88), compared to the parental S1 cells. Poziotinib did not significantly alter the efficacy (i.e., RF values) of mitoxantrone or SN-38 in the parental S1 cells, which do not express the ABCG2 transporter (Table 1). In contrast, the efficacy of mitoxantrone and SN-38 was significantly increased by 0.1, 0.3 or 0.6 μM of poziotinib in the S1-M1-80 cells (Table 1). Ko143, an inhibitor of ABCG2 transporter, did not significantly alter the efficacy of mitoxantrone or SN-38 in the parental S1 cells, whereas it significantly enhanced the efficacy of these anticancer drugs in the S1-M1-80 cells. Furthermore, 0.6 μM of poziotinib produced a decrease in resistance to mitoxantrone and SN-38 in the S1-M1-80 cells that was similar to that of 0.6 μM of Ko143 (Table 1). Finally, no significant difference was demonstrated in the IC_50_ values for oxaliplatin between the S1 and S1-M1-80 cells lines and neither poziotinib nor Ko143 significantly the RF value for oxaliplatin, which is not a substrate for the ABCG2 transporter.

The ABCB1-overexpressing SW620/Ad300 colon cancer cells were significantly resistant to paclitaxel (RF = 77.30) and doxorubicin (RF = 74.76) compared to the parental SW620 colon cancer cells that do not express the ABCB1 transporter (Table 2). Poziotinib did not significantly alter the IC_50_ values of paclitaxel or doxorubicin in the parental SW620 cells (Table 2). In contrast, poziotinib significantly increased the efficacy of doxorubicin and paclitaxel in the doxorubicin-resistant SW620/Ad300 cells (Table 2). Poziotinib, at 0.6 μM, decreased the RF values of paclitaxel and doxorubicin from 77.30- and 74.76-fold to 1.19- and 1.56-fold, respectively (Table 2). Verapamil, an inhibitor of the ABCB1 transporter, had no significant effect on the efficacy of paclitaxel or doxorubicin in the parental SW620 cells (Table 2). However, verapamil, at 0.6 μM, significantly increased the efficacy of paclitaxel (RF decreased from 77.30 to 3.79) and doxorubicin (RF decreased from 74.76 to 3.00) in the SW620/Ad300 cells (Table 2). Notably, the decrease in the RF values produced by 0.6 μM of poziotinib was significantly greater than that of 0.6 μM of verapamil (Table 2), indicating that poziotinib is more potent than verapamil in reversing MDR. There was no significant difference in the IC_50_ values of oxaliplatin between the SW620 and the SW620/Ad300 cells (Table 2). The RF value for oxaliplatin, which is not a substrate for the ABCB1 transporter, was not significantly altered by either poziotinib or verapamil in the SW620 and SW620/Ad300 cells (Table 2).

It has been shown that mutations at residue 482 in the ABCG2 transporter protein can affect the substrate transport profile and the efficacy of reversal compounds. Therefore, we used wild-type and mutant ABCG2 cell lines to further ascertain the reversal efficacy of poziotinib in transfected cells overexpressing mutant forms of the ABCG2 transporter. The transfection of HEK293 cells with the genes coding for the ABCG2-wild type (WT), ABCG2-R482G, and ABCG2-R482T mutant proteins, significantly decreased the efficacy of mitoxantrone and SN-38 compared to HEK293 cells transfected with an empty pcDNA3.1 vector (Table 3). It should be noted that the RF values for the S1-M1-80 colon cancer cells lines for mitoxantrone and SN-38 were significantly greater than for the HEK293 cells transfected with the ABCG2 gene. Poziotinib produced a significant increase in the efficacy of mitoxantrone and SN-38, as indicated by the decrease in the RF values, that were comparable to that of the ABCG2 inhibitor, Ko143 (Table 3). The IC_50_ values of oxaliplatin for the HEK293 cells transfected with the ABCG2 genes were not significantly altered by either poziotinib or Ko143.

The transfection of HEK293 cells with the ABCB1 transporter gene significantly decreased the efficacy of paclitaxel (RF = 23.20) and doxorubicin (RF = 25.66) compared to HEK293 cells transfected with an empty pcDNA3.1 vector (Table 4). The incubation of HEK293/ABCB1 cells with poziotinib or verapamil significantly decreased the resistance of paclitaxel and doxorubicin (Table 4). 

There was no significant difference in the IC_50_ values for oxaliplatin between the HEK293/pcDNA3.1 and HEK293/ABCB1 transfected cells (Table 4). Finally, as expected, neither poziotinib nor verapamil significantly altered the IC_50_ values of the non-ABCB1 substrate, oxaliplatin (Table 4).

### 2.3. Poziotinib Significantly Downregulates the ABCG2 but Not the ABCB1 Transporter Protein Expression Level Without Affecting the Membrane Localization of the ABCG2 and ABCB1 Transporters

**Figure 2:**
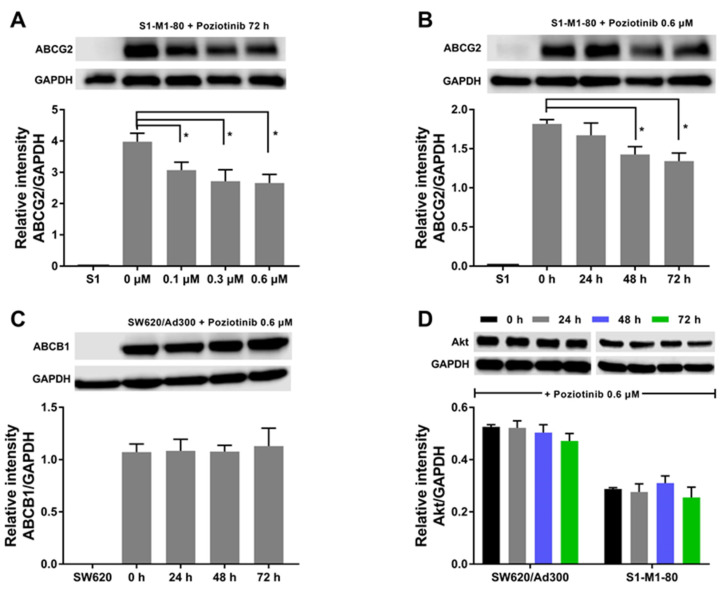
The effect of poziotinib on the expression level of the ABCB1 and ABCG2 transporter protein. (**A**) The effect of poziotinib on the expression level of ABCG2 in S1 and S1-M1-80 cells after 72 h of incubation (**B**) The effect of 0.1, 0.3 and 0.6 μM of poziotinib on the expression level of the ABCG2 protein in S1 and S1-M1-80 colon cancer cells after 72 h of incubation. (**C**) The effect of poziotinib on the expression level of ABCB1 transporter protein in SW620 and SW620/Ad300 colon cancer cells after 72 h of incubation. (**D**) The effect of poziotinib on the expression level of Akt in SW620/Ad300 and S1-M1-80 colon cancer cells after 72 h of incubation. Data are expressed as mean ±SD derived from three independent experiments. * *p* < 0.05 versus the control group. The uncropped western blots figures are shown in Figure S1.

It is possible that poziotinib’s increase in the efficacy of the chemotherapeutic drugs in the drug-resistant colon cancer cells could be due to downregulating the expression level of the ABCG2 and ABCB1 transporter protein. Therefore, we conducted western blotting experiments to evaluate the effect of poziotinib on the ABCG2 and ABCB1 protein expression level. Poziotinib, at 0.1, 0.3 and 0.6 μM, produced a significant decrease in the expression level of the ABCG2 transporter protein in S1-M1-80 cells after 72 h of incubation, compared to cells incubated with vehicle (0 μM) (Figure 2A). 

Furthermore, ABCG2 protein expression was significantly decreased in S1-M1-80 cells following incubation with 0.6 μM of poziotinib for 48 or 72 h (Figure 2B). In contrast, 0.6 μM of poziotinib did not significantly alter the expression level of the ABCB1 protein in SW620/Ad300 cells after 24, 48 or 72 h of incubation, compared to vehicle (Figure 2C). In addition, the expression level of the ABCG2 transporter can be modulated by the PI3K/Akt pathway, a downstream signaling pathway of EGFR. Consequently, we evaluated the effect of poziotinib on the expression of Akt. As shown in Figure 2D, poziotinib did not significantly affect the expression level of Akt, suggesting the poziotinib does not significantly alter Akt levels, which play a role in activating downstream pathways that mediate cancer cell proliferation [41].

**Figure 3:**
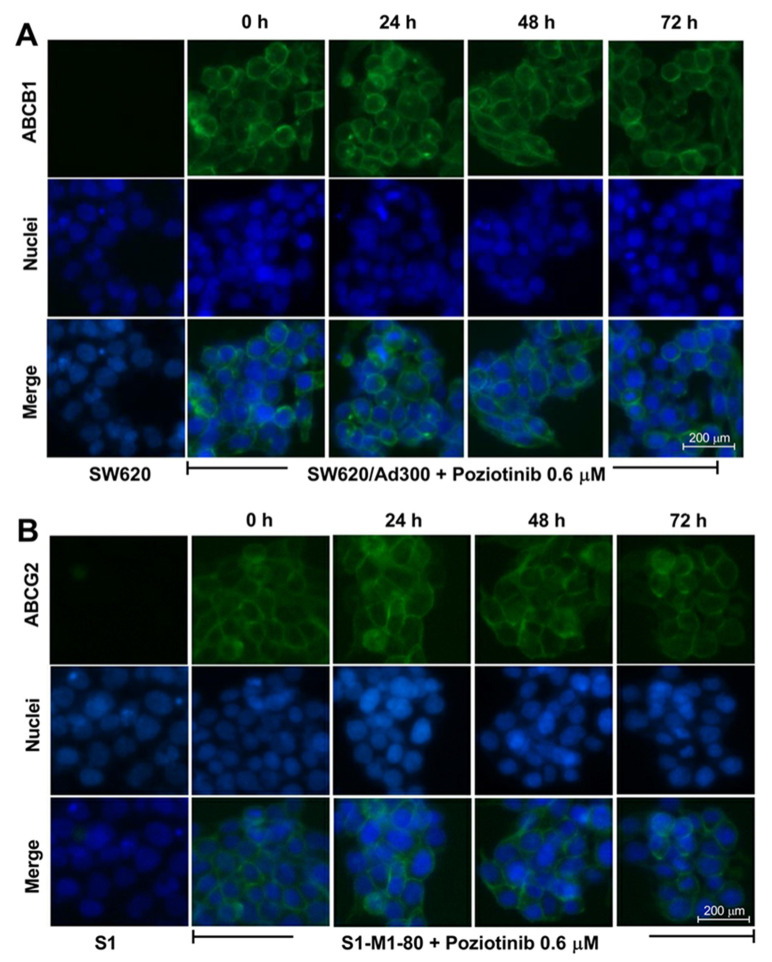
The effect of poziotinib on the membrane localization of the ABCB1 and ABCG2 transporters. (**A**) Cellular membrane localization of the ABCB1 transporter in SW620/Ad300 colon cancer cells incubated with 0.6 μM of poziotinib for 24, 48 or 72 h (**B**) Cellular membrane localization of the ABCG2 transporter in S1-M1-80 colon cancer cells incubated with 0.6 μM of poziotinib for 24, 48 or 72 h.

An alteration in the membrane localization of the ABCG2 and ABCB1 transporters (i.e., the transporters would not be located in the cell cytoplasmic membrane and thus can not efflux the anticancer drugs from the colon cancer cells) could decrease the resistance to the anticancer drugs. Consequently, we used an immunofluorescence assay to determine if poziotinib altered the membrane localization of the ABCG2 and ABCB1 transporters in drug-resistant S1-M1-80 and SW620/Ad300 colon cancer cells, respectively. The incubation of SW620/Ad300 or S1-M1-80 cells with 0.6 μM of poziotinib for 24, 48 or 72 h did not significantly the fluorescence intensity compared to cells incubated with vehicle (Figure 3A,B). These data suggest that poziotinib’s reversal of drug resistance in S1-M1-80 and SW620/Ad300 cells is not due to an alteration in the membrane localization of the ABCG2 and ABCB1 transporters.

### 2.4. Poziotinib Increases [^3^H]- Mitoxantrone Accumulation in S1-M1-80 Colon Cancer Cells by Downregulating the Expression of the ABCG2 Transporter Protein

**Figure 4:**
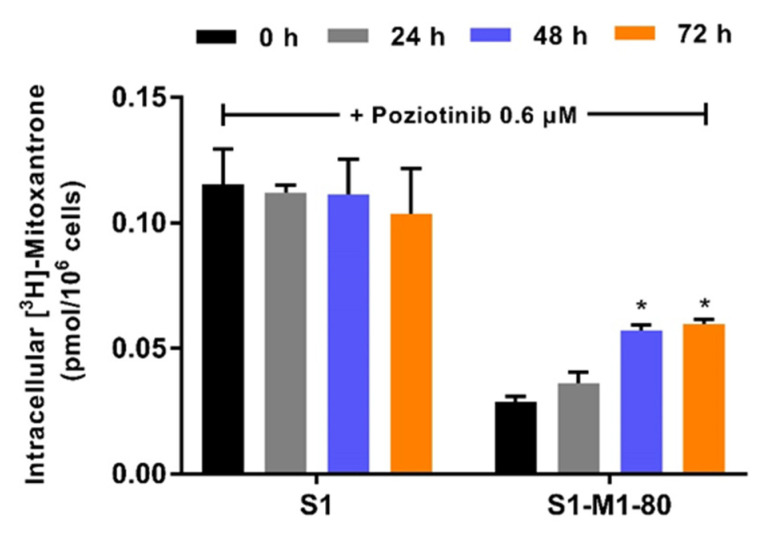
The effect of poziotinib on [^3^H]-mitoxantrone accumulation in S1 and S1-M1-80 colon cancer cells. The intracellular accumulation of [^3^H]-mitoxantrone was determined in S1 and S1-M1-80 colon cancer cells after incubation with 0.6 μM of poziotinib for 0, 24, 48 or 72 h. Data are expressed as mean ±SD derived from three independent experiments. * *p* < 0.05 versus the control group.

As indicated above, poziotinib (0.6 μM for 48 or 72 h) significantly downregulated the expression of the ABCG2 transporter protein in S1-M1-80 colon cancer cells. Subsequently, to validate this finding, we determined the effect of poziotinib on the accumulation of [^3^H]-mitoxantrone in S1 parental and S1-M1-80 resistant cells after 24, 48 or 72 h. The accumulation of [^3^H]-mitoxantrone in the ABCG2-overexpressing S1-M1-80 cells was 5-fold lower than that of the parental S1 cells. The incubation of parental S1 cells, which do not overexpress the ABCG2 transporter, with 0.6 μM of poziotinib for 24, 48 or 72 h did not significantly alter [^3^H]-mitoxantrone accumulation (Figure 4). In contrast, 0.6 μM of poziotinib significantly increased the accumulation of [^3^H]-mitoxantrone in S1-M1-80 cells after 48 and 72 h of incubation (Figure 4). Thus, the reversal of ABCG2-mediated resistance in S1-M1-80 cells can be, in part, due to a decrease in the expression of the ABCG2 transporter.

### 2.5. Poziotinib Significantly Stimulates ABCB1 and ABCG2 ATPase Activity

**Figure 5:**
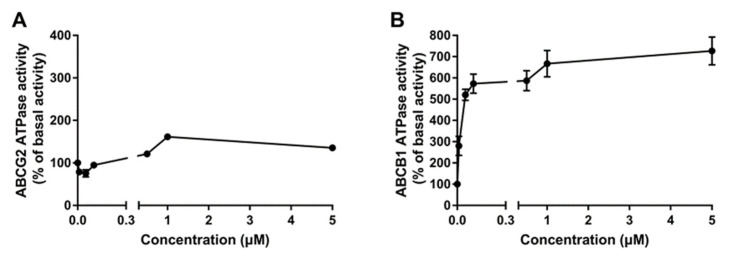
Poziotinib significantly stimulates ABCG2 and ABCB1 ATPase activity. (**A**) The effect of poziotinib on the ATPase activity of the ABCG2 transporter. (**B**) The effect of poziotinib on the ATPase activity of the ABCB1 transporter. Data are expressed as the mean ± SD derived from three independent experiments.

The drug efflux function of the ABCB1 and ABCG2 transporters has been reported to be linked to ATP hydrolysis, which can be stimulated or inhibited by ABCB1 and ABCG2 substrates, including the anticancer drugs used in this study. If a test compound stimulates ABCB1 or ABCG2 ATPase activity, this suggests that the compound is interacting with the transporter at the drug-substrate binding site. Consequently, we determined the effect of various concentrations of poziotinib on ABCB1 and ABCG2 transporter ATPase activity. Poziotinib produced a maximal increase of 1.65-fold in the basal activity of the ABCG2 transporter ATPase and the concentration required to produce a 50% increase (EC_50_) was 0.21 μM (Figure 5A). Interestingly, the two lowest concentrations of poziotinib decreased the basal activity of the ABCG2 ATPase (Figure 5A). In contrast, poziotinib produced a maximal increase of 7.27-fold in the basal activity of the ABCB1 transporter ATPase and the EC_50_ was 0.02 μM (Figure 5B). The stimulation of the ABCG2 and ABCB1 transporter ATPase activity by poziotinib suggests that it may interact with the transporters at the drug-substrate binding site.

### 2.6. Poziotinib Significantly Increases the Intracellular Accumulation of ABCG2 and ABCB1 Transporter Substrates in Colon Cancer Cells Overexpressing the ABCG2 and ABCB1 Transporter

**Figure 6:**
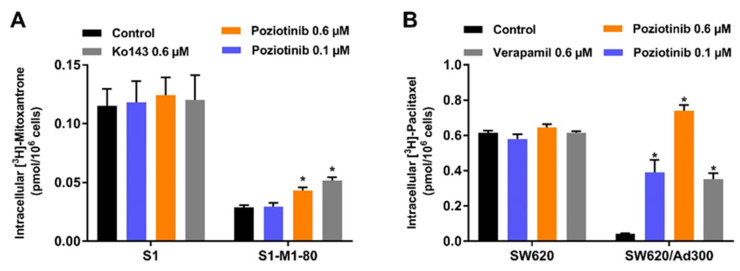
Poziotinib increases the accumulation of substrate drugs in MDR cells. (**A**) The effect of poziotinib on the intracellular accumulation of [^3^H]-mitoxantrone in S1 and S1-M1-80 colon cancer cells after 2 h of incubation with [^3^H]-mitoxantrone (**B**) The effect of poziotinib on the intracellular accumulation of [^3^H]-paclitaxel in SW620 and SW620/Ad300 colon cancer cells after 2 h of incubation with [^3^H]-paclitaxel. Ko143 and verapamil were used as positive controls (i.e., inhibitors) of the ABCG2 and ABCB1 transporters, respectively. Data are expressed as the mean ±SD based on three independent experiments. * *p* < 0.05 versus the control group.

The reversal of MDR in colon cancer cells overexpressing the ABCG2 or ABCB1 transporters could result from poziotinib inhibiting the efflux or transport function. Hence, we evaluated the effect of poziotinib on the intracellular accumulation of [^3^H]-mitoxantrone and [^3^H]-paclitaxel, which are substrates for the ABCG2 and ABCB1 transporters, respectively. As shown in Figure 6A, the intracellular accumulation of [^3^H]-mitoxantrone was significantly decreased in the drug-resistant, ABCG2 overexpressing S1-M1-80 cells compared to the parental S1 cells (Figure 6A). However, [^3^H]-mitoxantrone levels in the S1-M1-80 cells were significantly increased following 2 h of incubation with 0.6 μM of poziotinib or Ko143 compared to cells incubated with vehicle (Figure 6A). In contrast, [^3^H]-mitoxantrone accumulation level was not significantly altered by incubation with either poziotinib (0.1 or 0.6 μM) or Ko143 (0.6 μM) in the parental S1 cells which do not express the ABCG2 transporter, compared to cells incubated with vehicle (Figure 6A). These results suggest that poziotinib may increase the accumulation of [^3^H]-mitoxantrone in S1-M1-80 cells by inhibiting the efflux function of the ABCG2 transporter.

The intracellular accumulation of [^3^H]-paclitaxel was significantly lower in the ABCB1-overexpressing SW620/Ad300 colon cancer cells compared to the parental SW620 cells, indicating that the overexpression of the ABCB1 transporter increases the efflux of the substrate, [^3^H]-paclitaxel (Figure 6B). The incubation of SW620/Ad300 cells with either poziotinib (0.1 and 0.6 μM) or 0.6 μM of verapamil significantly increased the intracellular levels of [^3^H]-paclitaxel compared to cells incubated with vehicle (Figure 6B). Furthermore, 0.6 μM of poziotinib was significantly more efficacious than 0.6 μM of verapamil in increasing the accumulation of [^3^H]-paclitaxel for SW620/Ad300 cells (Figure 6B). Finally, the accumulation of [^3^H]-paclitaxel in the parental SW620 cells, which do not express the ABCB1 transporter, was not significantly altered by either poziotinib or verapamil compared to cells incubated with vehicle (Figure 6B).

### 2.7. Poziotinib Decreases the Efflux of the ABCB1 and ABCG2 Substrates, [^3^H]-Mitoxantrone and [^3^H]-Paclitaxel, Respectively, from MDR Colon Cancer Cells

**Figure 7:**
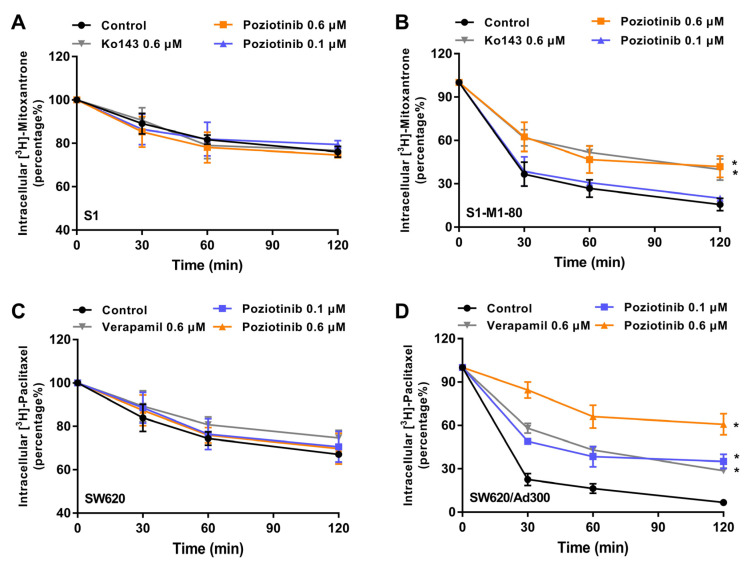
Poziotinib inhibits the efflux of the ABCG2 and ABCB1 substrates, [^3^H]-mitoxantrone and [^3^H]-paclitaxel, respectively, from MDR colon cancer cells. (**A**) The effect of poziotinib on the efflux of [^3^H]-mitoxantrone from S1 parental colon cancer cells. (**B**) The effect of poziotinib on the efflux of [^3^H]-mitoxantrone from S1-M1-80 colon cancer cells that overexpress the ABCG2 transporter. (**C**) The effect of poziotinib on the efflux of [^3^H]-paclitaxel from SW620 parental colon cancer cells. (**D**) The effect of poziotinib on the efflux of [^3^H]-paclitaxel from SW620/Ad300 colon cancer cells that overexpress the ABCB1 transporter. Ko143 was used as a positive control for inhibition of the ABCG2 transporter and verapamil was used as a positive control for inhibition of the ABCB1 transporter. Data are expressed as the mean ±SD based on three independent experiments. * *p* < 0.05 versus the control group.

It is possible that the increase in the accumulation of [^3^H]-mitoxantrone and [^3^H]-paclitaxel produced by poziotinib could result from it increasing substrate entry and/or decreasing substrate efflux. Therefore, we determined the effect of poziotinib on the efflux of [^3^H]-mitoxantrone and [^3^H]-paclitaxel from MDR colon cancer cells. The incubation of parental S1 cells, with vehicle for 0.5, 1 or 2 h, produced a 10, 20 and 25% decrease, respectively, in the intracellular levels of [^3^H]-mitoxantrone (Figure 7A). 

The efflux of [^3^H]-mitoxantrone from parental S1 cells was not significantly altered by incubating cells for 0.5, 1 or 2 h with 0.1 or 0.6 μM of poziotinib or 0.6 μM of Ko143 (Figure 7A), as these cells do not overexpress the ABCG2 transporter (Figure 7A). In contrast, in the presence of vehicle, the decrease in the intracellular levels of [^3^H]-mitoxantrone after 0.5, 1 or 2 h, was 65%, 75% and 85%, respectively, in the drug resistant S1-M1-80 cells, which overexpress the ABCG2 transporter (Figure 7B). These results indicate that the drug resistant S1-M1-80 cells efflux a significantly greater amount of [^3^H]-mitoxantrone than the parental S1 cells. The efflux of [^3^H]-mitoxantrone from the S1-M1-80 cells after 0.5 h of incubation was not significantly altered by 0.1 μM of poziotinib or 0.6 μM of Ko143 (Figure 7B). In contrast, 0.6 μM of poziotinib or 0.6 μM of Ko143 decreased the efflux of [^3^H]-mitoxantrone at 1 (from 65 to 40%) and 2 (from 75 to 50%) h in S1-M1-80 colon cancer cells compared to vehicle (Figure 7B). Thus, poziotinib, at 0.6 μM, and at the 1 and 2 h incubation periods, decreases the efflux of the ABCG2 substrate, [^3^H]-mitoxantrone, from ABCG2 overexpressing S1-M1-80 cells.

In the presence of vehicle for 0.5, 1 or 2 h, the efflux of the ABCB1 substrate, [^3^H]-paclitaxel, from SW620 parental colon cancer cells, was 10, 25 and 35%, respectively (Figure 7C). The efflux of [^3^H]-paclitaxel from parental SW620 cells, which do not overexpress the ABCB1 transporter, was not significantly altered by incubating cells for 0.5, 1 or 2 h with 0.1 or 0.6 μM of poziotinib or 0.6 μM of verapamil (Figure 7C). However, in the ABCB1 overexpressing SW620/Ad300 colon cancer cells incubated with vehicle, the efflux of [^3^H]-paclitaxel was 80, 85 and 95%, respectively (Figure 7D). Poziotinib, at 0.1 μM, decreased the efflux of [^3^H]-paclitaxel at 0.5 (from 80 to 50%), 1 (from 85 to 65%) and 2 h (from 95% to 60%) in SW620/Ad300 cells compared to vehicle (Figure 7D). Furthermore, 0.6 μM of poziotinib decreased the efflux of [^3^H]-paclitaxel at 0.5 (from 80 to 15%), 1 (from 85 to 35%) and 2 h (from 95 to 40%), respectively, compared to cells incubated with vehicle (Figure 7D). Similarly, the decrease in the efflux of [^3^H]-paclitaxel by 0.6 μM of verapamil at 0.5, 1 or 2 h was comparable to that produced by 0.1 μM of poziotinib (Figure 7D), indicating that poziotinib is more potent than verapamil in inhibiting the efflux of [^3^H]-paclitaxel by the ABCB1 transporter.

### 2.8. Poziotinib Reversiblely Inhibits the Efflux Function of the ABCG2 and ABCB1 Transporters

**Figure 8:**
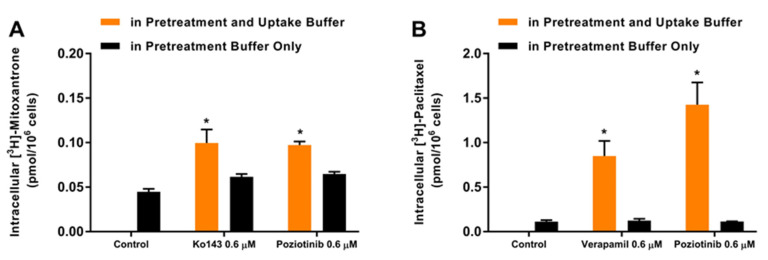
Poziotinib reversibly inhibits the efflux function of the ABCG2 and ABCB1 transporters. (**A**) The intracellular accumulation of [^3^H]-mitoxantrone in S1-M1-80 colon cancer cells after 2 h of preincubation with either vehicle (Control), Ko143 or poziotinib (**B**) The intracellular accumulation of [^3^H]-paclitaxel in SW620/Ad300 colon cancer after 2 h of preincubation with either vehicle (Control), verapamil or poziotinib. Ko143 was used as a positive control for inhibition of the ABCG2 transporter and verapamil was used as a positive control for inhibition of the ABCB1 transporter. Data are expressed as the mean ±SD based on three independent experiments. * *p <* 0.05 versus the control group.

In order to determine if pozitionib’s inhibition of the efflux function of the ABCG2 and ABCB1 transporters is reversible or irreversible inhibition, we conducted an accumulation assay. Both S1-M1-80 and SW620/Ad300 colon cancer cells were incubated in a pretreatment buffer with 0.6 μM of either poziotinib or Ko143 for 2 h. Subsequently, the cells were washed and incubated in an uptake buffer with [^3^H]-paclitaxel (an ABCB1 substrate) or [^3^H]-mitoxantrone (an ABCG2 substrate) with or without an inhibitor. The reversibility of the inhibitors was assessed by comparing the intracellular accumulation level of [^3^H]-paclitaxel and [^3^H]-mitoxantrone. As shown in Figure 8A, the intracellular accumulation of [^3^H]-mitoxantrone was significantly increased in S1-M1-80 colon cancer cells when 0.6 μM of poziotinib or Ko143 were present in the pretreatment and uptake buffer, a finding that was consistent with our above-mentioned results. In contrast, the accumulation of [^3^H]-mitoxantrone was not significantly altered when only poziotinib or Ko143 were presentin the pretreatment buffer (Figure 8A). Ko143 is a reversible ABCG2 inhibitor and its inhibition efficacy decreases following repeated washings [42]. These results suggest that poziotinib reversibly inhibits the efflux function of the ABCG2 transporter.

Similarly, the intracellular accumulation of [^3^H]-paclitaxel in SW620/Ad300 colon cancer cells was significantly increased when 0.6 μM of poziotinib or verapamil were present in the pretreatment and uptake buffer (Figure 8B). When poziotinib or verapamil were present in the pretreatment buffer, the intracellular accumulation of [^3^H]-paclitaxel was not significantly altered. Verapamil is a reversible ABCB1 inhibitor and its MDR reversal efficacy is abrogated immediately after washout [43], indicating poziotinib also reversibly inhibits the efflux function of the ABCB1 transporter.

### 2.9. Poziotinib Binds to the Drug-Substrate Pocket in the Human ABCB1and ABCG2 Transporters

**Figure 9:**
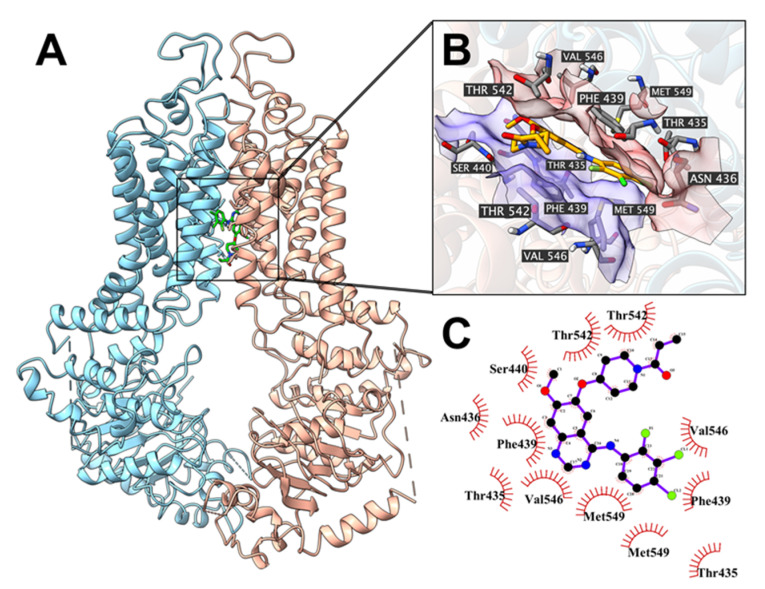
The interactions between poziotinib and the human ABCG2 transporter protein model. (**A**) An overview of the best-scoring pose for poziotinib in the drug-substrate site of the ABCG2 protein model (PDB: 6VXI). ABCG2 is displayed as colored tubes and ribbons (chain A: red; chain B: cyan). Poziotinib is displayed as colored sticks. (**B**) Details of interactions between poziotinib and ABCG2 protein drug-substrate binding site. ABCG2 helices are displayed as colored ribbons. Important residues are displayed as colored sticks (carbon: grey; oxygen: red; nitrogen: blue; hydrogen: white). The surface formed by important residues are depicted as solid planes (chain A: red; chain B: purple). Poziotinib is displayed as colored sticks (carbon: orange; oxygen: red; nitrogen: blue; chloride: lime; fluoride: light green). (**C**) A 2D diagram of the interaction between poziotinib and the ABCG2 transporter. The important amino acids are displayed as red arcs or dots/sticks.

Docking analysis experiments indicated that poziotinib could be docked into the drug-substrate binding site of the ABCG2 transporter, with a docking score of −10.1 kcal/mol. The interaction between poziotinib and important residues of ABCG2 is presented in Figure 9. 

The primary factor contributing to the binding of poziotinib to the ABCG2 transporter was hydrophobic interactions. Poziotinib is positioned and stabilized in the hydrophobic cavity, with surrounding residues, including Val546, Met549, Phe439, Thr435, Asn436, Thr542, Leu539, in chain A, and Val546, Met549, Phe439, Thr435, Ser440, Thr542, in chain B.

**Figure 10:**
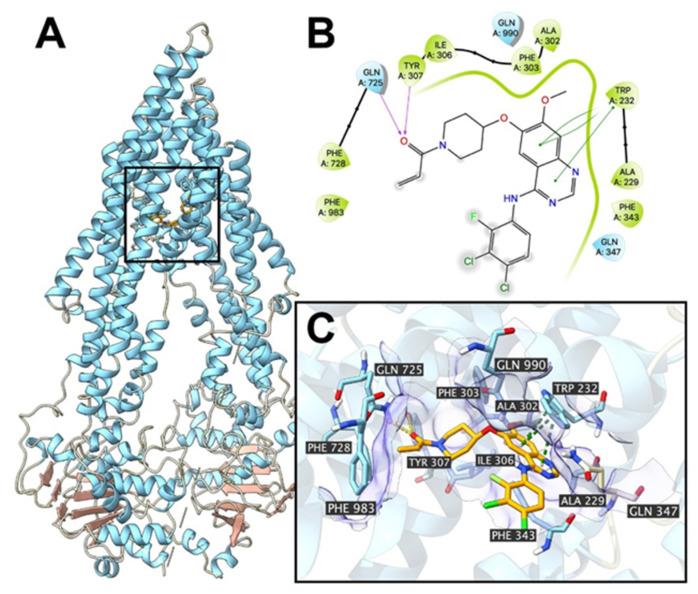
The interaction between poziotinib and human ABCB1 protein model. (**A**) An overview of the best-scoring pose for poziotinib in the drug-substrate binding site of the ABCB1 transporter protein (6QEX). ABCB1 is displayed as colored ribbons (helix: blue; strand: red; coil: white). Poziotinib was displayed as colored sticks. (**B**) A 2D diagram of the interactions between poziotinib and ABCB1. Amino acids within 3 Å from the ligand are displayed as colored bubbles (green: hydrophobic; blue: polar). Purple solid lines with arrows indicate hydrogen bonds. Green solid lines without arrows indicate π-π stacking interactions. (**C**) Details of interactions between poziotinib and the ABCB1 (6QEX) drug-substrate binding site. ABCB1 helices are displayed as colored ribbons. The important residues are displayed as colored sticks (carbon: orange; oxygen: red; nitrogen: blue; hydrogen: white; fluoride: lime; chloride: light green). The surface formed by the important residues are depicted as purple solid planes. Poziotinib are displayed as colored sticks (carbon: orange; oxygen: red; nitrogen: blue; chloride: lime; fluoride: light green).

Poziotinib was also shown to interact with the ABCB1 drug-substrate binding site and the docking score was -6.6 kcal/mol, which was lower than that for the ABCG2 transporter. Figure 10 illustrates molecular interactions between poziotinib and the ABCB1 transporter. The primary factor contributing to the binding of poziotinib to the ABCB1 protein was hydrophobic interactions. Poziotinib was positioned and stabilized in the hydrophobic cavity formed by Ala229, Ala302, Trp232, Ile306, Tyr307, Gln347, Phe343, Phe303 and Phe983. Also, poziotinib was stabilized by hydrogen bonds formed between Gln725 and Tyr307. Furthermore, the quinazoline structure was stabilized by stacking interactions formed with Trp232.

## 3. Discussion

Our results indicated that, in vitro, poziotinib, 0.1, 0.3 or 0.6 μM, demonstrated no significant cytotoxic effect to any of the cell lines we used. Therefore, we determined the effect of these concentrations of poziotinib on the efficacy of the ABCB1 and ABCG2 transporter anticancer drugs, paclitaxel and doxorubicin and mitoxantrone and SN-38, respectively, in the parental and drug-resistant colon cancer cells and HEK293 cells overexpressing the ABCG2 or ABCB1 transporter.

In this study, we determined the efficacy of poziotinib to overcome ABCG2- or ABCB1-mediated MDR in colon cancer cells and in HEK293 cells. We used two MDR colon cancer cell lines, S1-M1-80 and SW620/Ad300. S1-M1-80 is a mitoxantrone-selected, ABCG2-overexpressing cell line that has high resistance to mitoxantrone, irinotecan and SN-38 [44]. In contrast, SW620/Ad300 is a doxorubicin-selected, ABCB1-overexpressing cell line that has high resistance to colchicine, doxorubicin and paclitaxel [45]. In addition, HEK293 cells transfected with either the *ABCG2* or *ABCB1* gene were also used, as unlike drug-selected cancer cells, which could become drug resistant due to numerous mechanisms or pathways, the gene-transfected cells should only be resistant to ABCG2 and ABCB1 substrates by overexpressing the ABCG2 or ABCB1 transporters.

One of the main findings of this study was that poziotinib concentration-dependently increased the efficacy of mitoxantrone and SN-38 in ABCG2-overexpressing S1-M1-80 colon cancer cells and *ABCG2* transfected HEK293/ABCG2 cells. In contrast, poziotinib did not significantly alter the efficacy of mitoxantrone or SN-38 in the parental S1 colon cancer cells, a result consistent with their non-drug resistant phenotype. In addition, the minimal concentration of poziotinib to significantly reverse ABCG2 transporter-mediated resistance was lower (0.3 μM) than that of other tyrosine kinase inhibitors, such as VS-4718 (3 μM [46]), bafetinib (3 μM, [47]). Furthermore, the decrease in the RF value produced by 0.6 μM of poziotinib was similar to that of the compound, Ko143, a known inhibitor of the ABCG2 transporter [48]. However, poziotinib did not significantly alter the efficacy of the anticancer drug, oxaliplatin, which is not an ABCG substrate [47], in the S1 or S1-M1-80 colon cancer cells.

It has been shown that mutations at residue 482 can produce conformational changes that affect the binding of drugs and substrates and the efflux capacity of the ABCG2 transporter [49]. The substitution of Thr or Gly for Arg at position 482 produces resistance to a number of anticancer drugs, including doxorubicin and SN-38 [50]. Our results showed that poziotinib produced a concentration-dependent increase in the efficacy of mitoxantrone and SN-38 in the HEK293 cells transfected with ABCG2 DNA containing the R482G and R482T mutations. Although the majority of ABCG2 reversal compounds have reversal efficacy regardless of the mutation at residue 482, certain TKIs may have reversal efficacy that s more selective for the ABCG2-WT or mutant variants. For example, venetoclax [23], AC220 [51] and novobiocin [52] have been reported to reverse ABCG2-WT-mediated MDR but have no significant effect on the MDR mediated by the ABCG2 mutant variants. This is in contrast to poziotinib, which completely reversed MDR in HEK293 cells transfected with the ABCG2-WT or ABCG2 mutant variants. Overall, our results indicate that, in vitro, poziotinib reverses MDR produced by specific mutations in the DNA coding for the ABCG2 protein.

In ABCB1-overexpressing SW620/Ad300 colon cancer cells, poziotinib also produce a concentration-dependent increase in the efficacy of paclitaxel and doxorubicin in the SW620/Ad300 but not in the SW620 parental colon cancer cells and its efficacy was greater than verapamil, an ABCB1 inhibitor [53]. Furthermore, the concentration of poziotinib required to reverse ABCB1 transporter-mediated resistance was lower than that for other tyrosine kinase inhibitors such as lapatinib (2.5 μM, [54]) and nilotinib (2.5 μM, [55]). As in the S1-M1-80 colon cancer cells, poziotinib did not significantly the efficacy of oxaliplatin compared to cells incubated with vehicle. Thus, the results indicated that poziotinib is reversing MDR in S1-M1-80 and SW620/Ad300 colon cancer cells by inhibiting the ABCG2 and ABCB1 transporter. Therefore, we conducted experiments to delineate or ascertain the mechanism of action of poziotinib.

We conducted experiments to determine the effect of poziotinib on efflux activity of the ABCB1 or ABCG2 transporters [56,57,58]. As previously reported, [^3^H]-mitoxantrone accumulation was significantly lower in the ABCG2 overexpressing S1-M1-80, compared to the S1 (non-drug resistant) colon cancer cells [59]. Poziotinib (0.6 μM) and Ko143 (0.6 μM), an ABCG2 inhibitor, significantly increased the intracellular accumulation of [^3^H]-mitoxantrone, a substrate for the ABCG2 transporter [47], in the S1-M1-80 colon cancer cells but did not significantly affect accumulation on the S1 parental cells. The effect of poziotinib is comparable to other reported TKIs, such as TAE684 and venetoclax [22,23]. [^3^H]-paclitaxel accumulation, as previously reported, was significantly decreased in the ABCB1 overexpressing SW620/Ad300 colon cancer cells compared to the non-drug resistant parental SW620 colon cancer cells [60]. Poziotinib significantly increased the accumulation of [^3^H]-paclitaxel in the SW620/Ad300 colon cancer cells compared to the SW620 colon cancer cells with higher efficacy than verapamil. As reported for other compounds that reverse ABCB1 transporter-mediated resistance, such as erdafitinib and tepotinib [24,25], poziotinib increased [^3^H]-paclitaxel accumulation in SW620/Ad300 colon cancer cells to a similar magnitude. Thus, these results indicate that poziotinib reverses resistance (increases the efficacy) to paclitaxel and doxorubicin by inhibiting the efflux function of the ABCB1 transporter, i.e., poziotinib is a dual inhibitor of the ABCG2 and ABCB1 transporter. Our findings are similar to those for the TKI that are dual inhibitor such as lapatinib, nilotinib and VS-4718 [46,54,55].

Given that poziotinib could have increased the accumulation of [^3^H]-mitoxantrone or [^3^H]-paclitaxel by increasing their entry and/or decreasing its efflux, we determined the effect of poziotinib on the efflux of [^3^H]-mitoxantrone or [^3^H]-paclitaxel in the S1 and S1-M1-80 and the SW620 and SW620/Ad300 colon cancer cells, respectively. Neither poziotinib nor Ko143 significantly altered [^3^H]-mitoxantrone efflux in the S1 parental colon cancer cells, a finding consistent with the non-resistant phenotype of these cells. The efflux of [^3^H]-mitoxantrone was not significantly altered by 0.1 μM of poziotinib in S1-M1-80 cells. However, poziotinib and Ko143, at 0.6 μM, inhibited the efflux of [^3^H]-mitoxantrone in the S1-M1-80 cells. The poziotinib-induced decrease in [^3^H]- mitoxantrone efflux was similar to that reported for other TKIs, such as M3814, CC-671 and ulixertinib [61,62] As previously reported [50], the efflux of [^3^H]-paclitaxel was significantly greater from SW620/Ad300 colon cancer cells, which overexpress the ABCB1 transporter, compared to the non-drug resistant parental SW620 colon cancer cells. The incubation of parental SW620 colon cancer cells with either 0.1 or 0.6 μM of poziotinib or 0.6 μM of verapamil, an ABCB1 inhibitor, did not significantly affect the efflux of [^3^H]-paclitaxel. These findings are consistent with the fact that these cells do not overexpress the ABCB1 transporter and thus, inhibition of the ABCB1 transporter would not be predicted to affect [^3^H]-paclitaxel efflux. As a dual inhibitor of ABCB1 and ABCG2, poziotinib significantly inhibits the efflux function of ABCB1 and ABCG2, which is similar to other dual inhibitors such as selonsertib [19], sitravatinib [63] and alectinib [64]. It should be note that 0.1 μM of poziotinib was able to inhibit the efflux activity, while other dual ABCB1-ABCG2 inhibitors produced either a lower decrease or no effect at 0.1 μM [19,54,55]. In addition, our results show that the inhibition effect of poziotinib is reversible, similar to ABCB1 inhibitor verapamil and ABCG2 inhibitor Ko143 [42,43]. Therefore, poziotinib may bind to the substrate-binding site of the ABCB1 and ABCG2 transporters in a reversible manner, thereby inhibiting the binding of other transported substrates. Overall, our results indicate that, in part, poziotinib reverses MDR in S1-M1-80 and SW620/Ad300 colon cancer cell by inhibiting the efflux of ABCG2 and ABCB1 substrates, respectively. This effect will increase the intracellular levels and thus the efficacy of anticancer drugs in colon cancer cells overexpressing ABCG2 and ABCB1 transporters, thereby reversing MDR.

It is also possible that poziotinib could reverse ABCG2- and ABCB1-mediated MDR by decreasing the expression of the proteins for these transporters [65]. Using western blotting analysis, our results indicated that the incubation of S1-M1-80 colon cancer cells for 48 h with 0.6 μM of poziotinib significantly decreased the expression level of the ABCG2 transporter protein. Similarly, it has been reported that other TKIs such as PD153035 [66] and gefitinib [67], decrease the expression of the ABCG2 protein. Thus, poziotinib’s increase in the efficacy of mitoxantrone and SN-38 in S1-M1-80 colon cancer cells is due to its inhibition of the efflux activity and protein expression of the ABCG2 transporter. Consistent with these findings, the accumulation of the ABCG2 substrate, [^3^H]-mitoxantrone, was significantly decreased in S1-M1-80 colon cancer cells after 48 and 72 h of incubation with 0.6 μM of poziotinib. These results suggest that the decrease in [^3^H]-mitoxantrone accumulation produced by poziotinib at 48 and 72 h could be due, in part, to the downregulation of the ABCG2 transporter. The mechanism(s) by which poziotinib produces a decrease in the expression of the ABCG2 protein remain to be elucidated. It could result from alterations in the transcription of the ABCG2 gene, mRNA translation and/or stability of the ABCG2 protein. In contrast, the expression of the ABCB1 protein in SW620/Ad300 colon cancer cells was not significantly altered by poziotinib. Therefore, poziotinib’s reversal of MDR in these cells is unlikely due to its effect on the expression of the ABCB1 protein. Although the EGFR signaling pathway may also regulate the expression of the ABCB1 transporter, poziotinib did not significantly affect the expression of the ABCB1 transporter protein. Hence, the EGFR signaling pathway may not contribute to poziotinib’s ABCB1 reversal efficacy. However, it remains to be determined if incubation periods greater than 72 h can decrease the expression of the ABCB1 protein.

Poziotinib could reverse MDR in the S1-M1-80 and SW620/Ad300 cells by causing the translocation of the ABCG2 and ABCB1 transporters, respectively, i.e., the transporters are no longer present on the cell membrane and thus cannot efflux anticancer drugs. However, this is unlikely as the incubation of the colon cancer cells with 0.6 μM of poziotinib for 72 h demonstrated no significant effect to the localization of the ABCG2 or ABCB1 transporters on cell membranes. These findings also suggest that there is no internalization of the ABCG2 or ABCB1 transporters from the cell membrane, at least after 72 h of incubation with 0.6 μM of poziotinib.

It has been suggested that the activation of EFGR can activate the downstream Akt pathway, which regulates the expression of ABCG2 and ABCB1 [41,68,69,70]. Given that poziotinib is an EGFR inhibitor [71], we evaluated the effect of poziotinib on the expression level of the Akt protein. Poziotinib, at 0.6 μM, did not significantly affect Akt protein levels, suggesting that the downregulation of ABCG2 protein expression is not due to alterations in Akt protein levels. However, poziotinib could affect the level of expression of other proteins in the Akt pathway, although this remains to be determined. It is possible that the antagonism of EGFR by poziotinib could contribute to the ABCG2 reversal efficacy of poziotinib. However, this is unlikely as both the parental and MDR colon cancer cell lines have similar expression levels of EGFR [57,58]. However, further studies must be conducted to evaluate the expression level of EGFR and its downstream proteins to support our aforementioned conclusion.

It has been shown that certain compounds can (1) inhibit the ATPase activity of ABC transporters, thus inhibiting the hydrolysis of ATP, which is critical for drug and substrate efflux [60,61,62] or (2) stimulate ATPase activity by binding to the substrate-drug binding site of the ABC transporters [20,72]. Our results indicated that poziotinib stimulated the ATPase activity of the ABCB1 and ABCG2 transporters. The stimulation of the ABCG2 and ABCB1 ATPase activity produced by poziotinib suggests that it interacts with the substrate-drug binding site and therefore may inhibit the binding of other substrates, including anticancer drugs, thus inhibiting their efflux by the ABCG2 or ABCB1 transporters. Poziotinib was more potent in activating the ATPase activity of the ABCB1, compared to the ABCG2 transporter. This could, in part, explain why poziotinib was more potent in reversing the resistance to the ABCB1 substrates, paclitaxel and doxorubicin, than the ABCG2 substrates, mitoxantrone and SN-38 in the colon cancer cells, although this remains to be elucidated. However, it should be pointed out that the ABCG2 and ABCB1 ATPase was expressed in High Five insect cells, whereas the effects of poziotinib on the resistance to the anticancer drugs were done in colon cancer cells.

Computational molecular docking analysis is an approach that has been widely used to predict the interaction of ligands with proteins [73]. Although this technique may not be indicative of the in vivo binding of a ligand to a protein, it can be used to identify compounds that interact with the substrate-drug binding site in ABC transporters [74,75]. Our docking analysis studies indicated that poziotinib interacted with the homology models of the ABCB1 (−6.6 kcal/mol) and ABCG2 (−10.1 kcal/mol) transporters. The lower docking score of poziotinib for the ABCG2 transporter compared to the ABCB1 transporter could be due to it forming a greater number of hydrophobic bonds with the ABCG2 transporter. In the ABCB1 docking analysis, the score of poziotinib was comparable to other ABCB1 reversal agents such as CGM-097 (−8.5 kcal/mol), erdafitinib (−8.5 kcal/mol) and verapamil (−7.376 kcal/mol) [25,76]. Poziotinib’s docking score was slighter higher than that for ABCG2 inhibitors, such as venetoclax (−12.1 kcal/mol) and sitravatinib (−13.248 kcal/mol) [23,75]. Furthermore, the docking results suggested that poziotinib interacts with the substrate-drug binding sites in the ABCG2 and ABCB1 proteins, a finding that supports our data indicating that poziotinib inhibits the efflux activity of these transporters.

In conclusion, our results show that poziotinib reverses MDR in ABCG2- and ABCB1-overexpressing colon cancer cells to their corresponding anticancer drug substrates, It is likely that poziotinib reverses MDR in ABCG2-overexpressing cells by inhibiting the efflux activity of the ABCG2 transporter, as well as by downregulating the expression level of the ABCG2 transporter protein. However, poziotinib reverses MDR in SW620/Ad300 colon cancer cells by only inhibiting the efflux activity of the ABCB1 transporter. It should be noted that ABC transporters are also expressed in normal cells to protect tissues and organs and therefore, it is important to develop methods to deliver MDR reversal compounds, as well as with anticancer drugs, only to cancer cells [10]. For example, nano drug co-delivery systems, which can load two or more anticancer drugs may be used to increase drug delivery to cancer cells and decrease the incidence of adverse effects [77,78]. In addition, single chain antibodies have been shown to increase the specificity and efficacy of certain drugs [79]. If our in vitro results can be translated to humans, they suggest that poziotinib, in combination with specific anticancer drugs such as irinotecan, SN-38 and 5-FU [30], could be used to combat ABCG2- and ABCB1-mediated MDR in patients with CRC.

## 4. Materials and Methods

### 4.1. Reagents

Poziotinib was acquired from MedChemExpress LLC (MCE, Monmouth Junction, NJ, USA), Ko143 was purchased from Enzo Life Sciences (Farmingdale, NY, USA). All other chemicals were purchased from Sigma Chemical Co (St. Louis, MO, USA) unless stated otherwise. Stock solutions (10 mM) were reconstituted in DMSO for all the drugs except oxaliplatin, which was dissolved in dimethylformamide.

### 4.2. Cell Lines and Cell Culture

The following cell lines were cultured as previously described [75]. Briefly, the colon cancer cell line SW620 and its ABCB1-overexpressing subline SW620/Ad300, S1 and its ABCG2-overexpressing subline S1-M1-80, gene-transfected HEK293, HEK293/ABCB1 and HEK293/ABCG2 cells, were maintained in DMEM with 10% FBS. S1-M1-80 cells were maintained in the presence of 80 μM of mitoxantrone [44]. SW620/Ad300 cells were maintained in the presence of 300 ng/mL of doxorubicin [45]. The transfected cell lines were maintained in medium with 2 mg/mL of G418. All cell lines were maintained in humid incubator (37 °C, 5% CO_2_) and subcultured at 80% confluency.

### 4.3. MTT Cell-Proliferation Assay

The cytotoxicity of the drug treatments were evaluated using the MTT cell-proliferation assay as previous described [24]. Briefly, cells were seeded in 96-well plates at the appropriate density in each well. The cells were then incubated with different combinations of the anticancer drugs and poziotinib. After 72 h of incubation, cell viability was assessed by further incubating the cells with the MTT solution for 4 h. DMSO (100 μL) was added to dissolve the resulting formazan crystals. The optical densities at 570 nm was measured using a Fisherbrand™ accuSkan™ GO UV/Vis Microplate Spectrophotometer (Thermo Fisher Scientific Inc., Waltham, MA, USA).

### 4.4. Immunoblotting

The immunoblots were performed with same protocol as previously described [61]. The antibodies used in this study are ABCG2, ABCB1, Akt or GAPDH (1:1000 dilution, Thermo Fisher Scientific Inc., Waltham, MA, USA) and anti-rabbit IgG HRP-linked or anti-mouse IgG HRP-linked antibodies (1:1000 dilution, Cell Signaling Technology Inc., Danvers, MA, USA). The immunoreactive bands were visualized using ECL substrate (Thermo Fisher Scientific Inc.), acquired by C-DiGit^®^ Blot Scanner with Image Studio V5.2 (LI-COR Biosciences, Lincoln, NE, USA), and analyzed by ImageJ software (NIH, Bethesda, MD, USA).

### 4.5. Immunofluorescence Microscopy

Cells were seeded in 24-well plates (2 × 10^5^ cells per well). The cells were incubated with 0.6 μM of poziotinib at 0, 24, 48, and 72 h. After incubation, the cells were fixed, permeabilized and blocked in PBS with 6% BSA (VWR Chemicals, LLC, Radnor, PA, USA). The ABCB1 and ABCG2 transporters were visualized using either an ABCB1 or ABCG2 antibody (1:1000, Thermo Fisher Scientific Inc.), followed by incubation with Alexa Fluor 488 conjugated anti-mouse IgG antibody (1:1000, Thermo Fisher Scientific Inc). The nuclei were stained using a DAPI solution. The immunoreactivity was visualized using a Nikon TE-2000S fluorescence microscope (Nikon Instruments Inc., Melville, NY, USA).

### 4.6. [^3^H]-Substrate Accumulation and Efflux Assay

Colon cancer cells were seeded in 24-well plates (2 × 10^5^ cells per well). The cells were incubated with or without poziotinib for 2 h at 37 °C. [^3^H]-paclitaxel was used as a substrate drug for SW620 and SW620/Ad300 colon cancer cells. [^3^H]-mitoxantrone was used as a substrate drug for S1 and S1-M1-80 colon cancer cells. In the substrate accumulation assay, cells were incubated with DMEM containing 5 nM of [^3^H]-paclitaxel or [^3^H]-mitoxantrone, in the presence or absence of a reversal compound, for 2 h. In the substrate efflux assay, the cells were further incubated without a [^3^H]-substrate, and in the presence or absence of reversal compounds, for 0, 30, 60 and 120 min. The determination of reversibility was performed using a previously described protocol, with modifications [43]. Finally, the samples were collected in scintillation vials, where scintillation fluid was added and the radioactivity was determined using a Packard TRICARB 1900CA liquid scintillation analyzer (Packard Instrument, Downers Grove, IL, USA).

### 4.7. Evaluation of ABCB1 and ABCG2 ATPase Activity

The effect of poziotinib on the ATPase activity of the ABCGB1 and ABCG2 transporters was determined as previously described [80].

### 4.8. In Silico Molecular Docking Analysis

The 3-D structure of poziotinib was constructed for docking simulations with the human ABCB1 (6QEX) [62] and ABCG2 (6VXI) [63] models obtained from RCSB Protein Data Bank, as previously described [23]. The docking grid center coordinates were determined from the bound ligand: (1) paclitaxel from the 6QEX PDB files and (2) mitoxantrone from the 6VXI PDB files. The docking calculations were performed using AutoDock Vina (version 1.1.2) [81]. The top-scoring poses (sorted based on the affinity score in kcal/mol) were selected for further analysis and visualization.

### 4.9. Statistical Analysis

Data were expressed as mean ± SD from three independent experiments and analyzed using GraphPad Prism 8.1 software (GraphPad, San Diego, CA, USA). Statistical analysis was performed using a one-way ANOVA. The significance value was indicated as * *p* < 0.05, ** *p* < 0.01, *** *p* < 0.005.

## 5. Conclusions

The results of our study indicate that poziotinib increases the efficacy of chemotherapeutic drugs (i.e., reverses MDR) in MDR colon cancer cells by interacting with and affecting the efflux activity of the ABCG2 and ABCB1 transporters and the protein expression level of the ABCG2 transporter. If our results can be translated to humans, they suggest a novel strategy of combining poziotinib with certain anticancer drugs to treat CRC patients with MDR cancer due to the overexpression of ABCG2 and/or ABCB1 transporters.

## Figures and Tables

**Table 1:** The effect of poziotinib and Ko143 on the efficacy of mitoxantrone, SN-38 and oxaliplatin in colon cancer cells overexpressing the ABCG2 transporter.

Drugs	IC_50_ Value ± SD ^a^ (μM, RF ^b^)	
	S1	S1-M1-80
Mitoxantrone	0.046 ± 0.006 (1.00)	5.768 ± 0.587 (125.75)
+Poziotinib 0.1 μM	0.042 ± 0.012 (0.92)	2.401 ± 0.486 (52.35) *
+Poziotinib 0.3 μM	0.045 ± 0.020 (0.99)	0.607 ± 0.225 (13.23) **
+Poziotinib 0.6 μM	0.045 ± 0.011 (0.98)	0.342 ± 0.165 (7.45) *
+Ko143 0.6 μM	0.050 ± 0.010 (1.09)	0.291 ± 0.144 (6.34) *
SN-38	0.060 ± 0.015 (1.00)	5.916 ± 0.783 (97.88)
+Poziotinib 0.1 μM	0.052 ± 0.008 (0.85)	2.847 ± 0.731 (47.11)
+Poziotinib 0.3 μM	0.068 ± 0.027 (1.12)	1.001 ± 0.143 (16.57) *
+Poziotinib 0.6 μM	0.069 ± 0.019 (1.14)	0.485 ± 0.012 (8.03) **
+Ko143 0.6 μM	0.086 ± 0.006 (1.42)	0.328 ± 0.110 (5.42) **
Oxaliplatin	4.091 ± 0.525 (1.00)	3.719 ± 0.622 (0.91)
+Poziotinib 0.1 μM	3.763 ± 0.467 (0.92)	4.061 ± 1.101 (0.99)
+Poziotinib 0.6 μM	5.325 ± 0.492 (1.30)	4.417 ± 1.083 (1.08)
+Ko143 0.6 μM	3.719 ± 0.294 (0.91)	4.780 ± 0.232 (1.17)

^a^ The half maximal inhibitory concentrations (IC_50_ values) are represented as the mean ± SD of at least three independent experiments performed in triplicate ^b^ RF: The resistance-fold value was calculated by dividing the IC_50_ values of the drugs in the S1-M1-80 colon cancer cells in the presence or absence of poziotinib or Ko143 by the IC_50_ of the S1 parental cells in the absence of poziotinib or Ko143. * *p* < 0.05, ** *p* < 0.01 versus the control group in the absence of poziotinib or Ko143.

**Table 2:** The effect of poziotinib and verapamil on the efficacy of paclitaxel, doxorubicin and oxaliplatin in colon cancer cells overexpressing the ABCB1 transporter.

Drugs	IC_50_ Value ± SD ^a^ (μM, RF ^b^)	
	SW620	SW620/Ad300
Paclitaxel	0.190 ± 0.033 (1.00)	14.680 ± 3.722 (77.30)
+Poziotinib 0.1 μM	0.179 ± 0.062 (0.94)	5.671 ± 0.469 (29.86) *
+Poziotinib 0.3 μM	0.190 ± 0.005 (1.00)	0.760 ± 0.181 (9.96) *
+Poziotinib 0.6 μM	0.168 ± 0.006 (0.88)	0.225 ± 0.012 (1.19) **
+Verapamil 0.6 μM	0.184 ± 0.012 (0.97)	0.719 ± 0.070 (3.79) **
Doxorubicin	0.187 ± 0.028 (1.00)	13.985 ± 2.170 (74.76)
+Poziotinib 0.1 μM	0.193 ± 0.040 (1.03)	6.596 ± 0.174 (35.26) *
+Poziotinib 0.3 μM	0.180 ± 0.021 (0.96)	1.065 ± 0.351 (5.69) **
+Poziotinib 0.6 μM	0.155 ± 0.007 (0.83)	0.291 ± 0.085 (1.56) ***
+Verapamil 0.6 μM	0.183 ± 0.021 (0.98)	0.561 ± 0.104 (3.00) ***
Oxaliplatin	12.484 ± 1.254 (1.00)	13.614 ± 1.336 (1.09)
+Poziotinib 0.1 μM	13.768 ± 0.924 (1.10)	12.908 ± 1.364 (1.03)
+Poziotinib 0.6 μM	11.708 ± 1.101 (0.94)	14.048 ± 1.968 (1.13)
+Verapamil 0.6 μM	11.378 ± 0.940 (0.91)	13.458 ± 2.634 (1.08)

^a^ The half maximal inhibitory concentration (IC_50_ values) are represented as the mean ±SD of at least three independent experiments performed in triplicate ^b^ RF: The resistance-fold value was calculated by dividing the IC_50_ values of drugs in the SW620/Ad300 colon cancer cells in the presence or absence of poziotinib or verapamil by the IC_50_ of the S1 parental cells in the absence of poziotinib or verapamil. * *p* < 0.05, ** *p* < 0.01, *** *p* < 0.005 versus the control group without poziotinib or verapamil.

**Table 3:** The effect of poziotinib and Ko143 on the efficacy of mitoxantrone, SN-38 and oxaliplatin in *ABCG2* gene-transfected HEK293 cells overexpressing the ABCG2 transporter.

Treatment	IC_50_ Value ± SD ^a^ (μM, RF ^b^)			
	pcDNA3.1	ABCG2-WT	ABCG2-R482G	ABCG2-R482T
Mitoxantrone	0.105 ± 0.021 (1.00)	2.551 ± 0.508 (24.01)	2.827 ± 0.672 (27.03)	2.197 ± 0.557 (21.01)
+Poziotinib 0.1 μM	0.122 ± 0.019 (1.17)	0.332 ± 0.018 (3.18) **	0.402 ± 0.020 (3.84) *	0.907 ± 0.137 (8.67) *
+Poziotinib 0.3 μM	0.098 ± 0.022 (0.94)	0.266 ± 0.079 (2.55) *	0.287 ± 0.074 (2.74) ***	0.423 ± 0.191 (4.04) *
+Poziotinib 0.6 μM	0.127 ± 0.027 (1.22)	0.150 ± 0.003 (1.44) *	0.273 ± 0.039 (2.61) *	0.200 ± 0.086 (1.91) **
+Ko143 0.6 μM	0.104 ± 0.026 (0.99)	0.232 ± 0.017 (2.21) *	0.146 ± 0.043 (1.39) **	0.177 ± 0.088 (1.69) **
SN-38	0.156 ± 0.050 (1.00)	3.905 ± 0.245 (25.07)	6.149 ± 0.582 (39.47)	3.543 ± 0.122 (22.75)
+Poziotinib 0.1 μM	0.203 ± 0.066 (1.31)	1.363 ± 0.190 (8.75) *	1.239 ± 0.208 (7.96) **	1.483 ± 0.200 (9.52) *
+Poziotinib 0.3 μM	0.206 ± 0.039 (1.32)	0.349 ± 0.120 (2.24) ***	0.556 ± 0.195 (3.57) **	0.646 ± 0.190 (4.15) ***
+Poziotinib 0.6 μM	0.198 ± 0.019 (1.27)	0.182 ± 0.044 (1.17) ***	0.226 ± 0.079 (1.45) ***	0.363 ± 0.132 (2.33) ***
+Ko143 0.6 μM	0.171 ± 0.038 (1.10)	0.338 ± 0.141 (2.17) **	0.194 ± 0.064 (1.25) **	0.605 ± 0.141 (3.88) ***
Oxaliplatin	7.004 ± 0.865 (1.00)	9.412 ± 2.000 (1.34)	6.321 ± 1.153 (0.90)	5.836 ± 0.221 (0.83)
+Poziotinib 0.1 μM	7.063 ± 0.984 (1.01)	7.057 ± 1.939 (1.01)	7.926 ± 1.839 (1.13)	6.219 ± 1.127 (0.89)
+Poziotinib 0.6 μM	5.672 ± 0.368 (0.81)	7.318 ± 0.890 (0.04)	6.638 ± 2.370 (0.95)	6.785 ± 0.382 (0.97)
+Ko143 0.6 μM	5.785 ± 0.374 (0.83)	8.259 ± 0.832 (1.18)	6.590 ± 1.544 (0.94)	6.358 ± 1.138 (0.91)

^a^ The half maximal inhibitory concentrations (IC_50_ values) are represented as the mean ± SD of at least three independent experiments performed in triplicate ^b^ The resistance-fold was calculated by dividing the IC_50_ values of drugs in the ABCG2-WT, ABCG2-R482G or ABCG2-R482T cells in the presence or absence of poziotinib or Ko143 by the IC_50_ of the HEK293/pcDNA3.1 cells (which do not express ABCG2) in the absence of poziotinib or Ko143. * *p* < 0.05, ** *p* < 0.01, *** *p* < 0.005 versus the control group without poziotinib or Ko143.

**Table 4:** The effect of poziotinib and verapamil on the efficacy of paclitaxel, doxorubicin and oxaliplatin in *ABCB1* gene-transfected HEK293 cells overexpressing the ABCB1 transporter.

Drugs	IC_50_ Value ± SD ^a^ (μM, RF ^b^)	
	HEK293/pcDNA3.1	HEK293/ABCB1
Paclitaxel	0.111 ± 0.006 (1.0)	2.569 ± 0.603 (23.20)
+Poziotinib 0.1 μM	0.126 ± 0.015 (1.14)	1.301 ± 0.159 (11.75) *
+Poziotinib 0.3 μM	0.120 ± 0.013 (1.08)	0.573 ± 0.164 (5.17) *
+Poziotinib 0.6 μM	0.104 ± 0.009 (0.94)	0.216 ± 0.082 (1.95) **
+Verapamil 0.6 μM	0.113 ± 0.011 (1.02)	0.499 ± 0.173 (4.51) *
Doxorubicin	0.076 ± 0.006 (1.00)	1.958 ± 0.651 (25.66)
+Poziotinib 0.1 μM	0.075 ± 0.005 (0.98)	0.750 ± 0.135 (9.83) *
+Poziotinib 0.3 μM	0.076 ± 0.013 (1.00)	0.361 ± 0.020 (4.74) **
+Poziotinib 0.6 μM	0.064 ± 0.002 (0.84)	0.204 ± 0.061 (2.67) ***
+Verapamil 0.6 μM	0.081 ± 0.002 (1.06)	0.288± 0.093 (3.77) **
Oxaliplatin	7.004 ± 0.865 (1.00)	6.981 ± 0.654 (1.00)
+Poziotinib 0.1 μM	7.063 ± 0.984 (1.01)	7.871 ± 2.158 (1.12)
+Poziotinib 0.6 μM	6.779 ± 0.357 (0.97)	8.128 ± 0.754 (1.16)
+Verapamil 0.6 μM	5.783 ± 0.374 (0.83)	7.211 ± 1.664 (1.03)

^a^ The half maximal inhibitory concentrations (IC_50_ values) are represented as the mean ± SD of at least three independent experiments performed in triplicate ^b^ The resistance-fold value was calculated by dividing the IC_50_ values of drugs in the HEK293/ABCB1 transfected cells (which overexpress ABCB1) in the presence or absence of poziotinib or verapamil by the IC_50_ of the HEK293/pcDNA3.1 cells (which do not express ABCB1) in the absence of poziotinib or verapamil. * *p* < 0.05, ** *p* < 0.01, *** *p* < 0.005 versus the control group without poziotinib or verapamil.

## Supplementary Materials

The following are available online at https://www.mdpi.com/2072-6694/12/11/3249/s1, Figure S1: The uncropped Western blots.
