# Supplementary material for: Poziotinib Inhibits the Efflux Activity of the ABCB1 and ABCG2 Transporters and the Expression of the ABCG2 Transporter Protein in Multidrug Resistant Colon Cancer Cells
Source: Cancers (Basel). 2020 Nov 4;12(11):3249. doi: 10.3390/cancers12113249 (PMC7694178; doi:10.3390/cancers12113249)
Supplement: Supplementary file 1 [file cancers-12-03249-s001.pdf]

cancers

Figure S1. The uncropped Western blots.

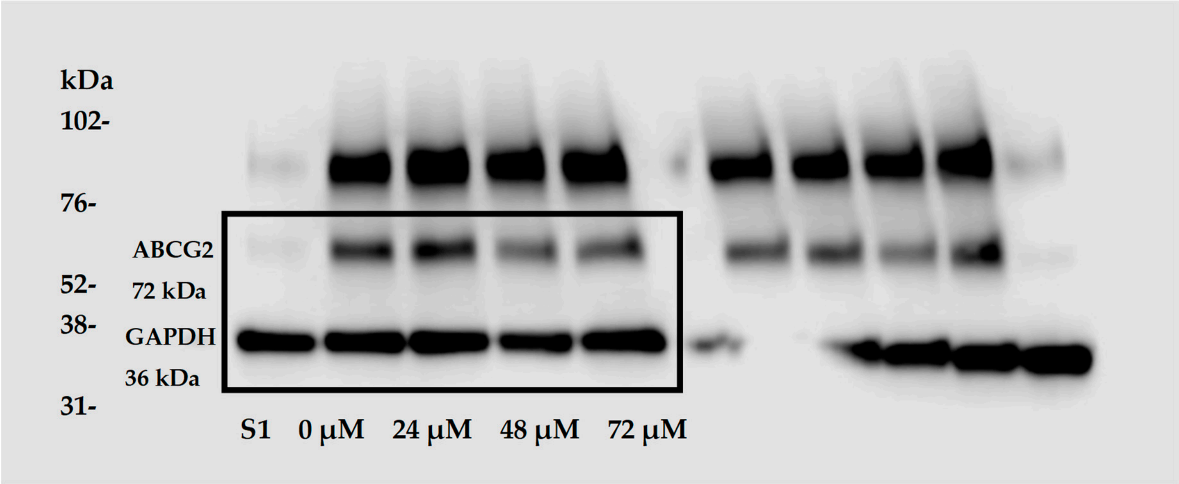

IntDen	S1	S1-M1-80 0 μM	S1-M1-80 0.1 μM	S1-M1-80 0.3 μM	S1-M1-80 0.6 μM
ABCG2	16593	198295	229660	147966	189444
GAPDH	314924	327081	338999	276195	361267
Relative intensity (%)	0.052689	0.606257	0.677465	0.53573	0.524388

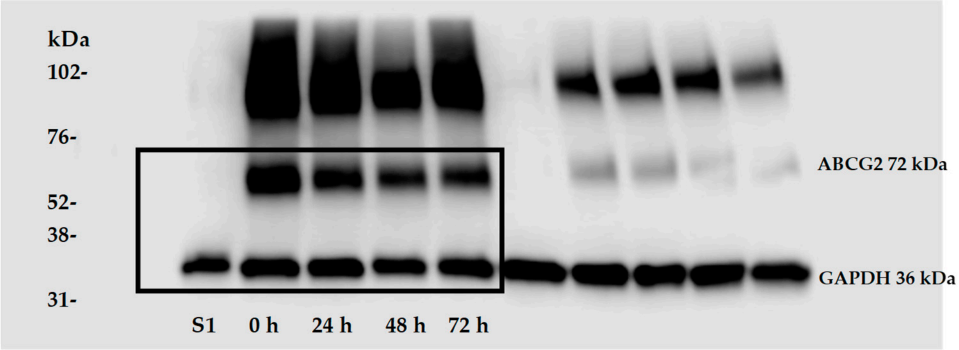

IntDen.	S1	S1-M1-80 0 h	S1-M1-80 24 h	S1-M1-80 48 h	S1-M1-80 72 h
ABCG2	4718	411080	298898	274869	291952
GAPDH	250490	322736	310642	267814	315014
Relative intensity (%)	0.018835	1.273735	0.962194	1.026343	0.926791

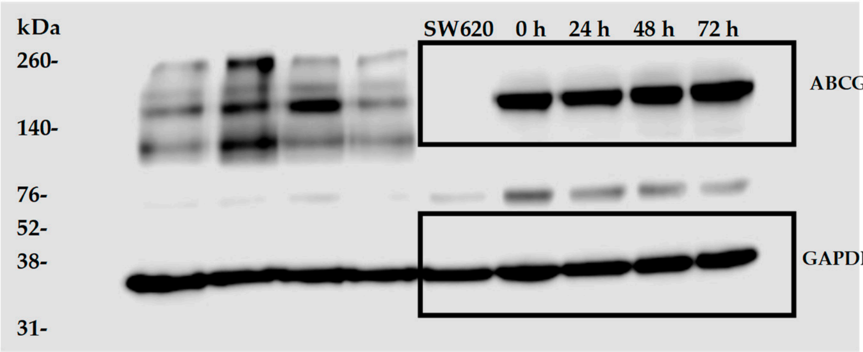

IntDen	SW620	SW620/Ad300 0 h	SW620/Ad300 24 h	SW620/Ad300 48 h	SW620/Ad300 72 h
ABCB1	35	340287	353994	351048	423318
GAPDH	320712	347497	346311	329213	340801
Relative intensity (%)	0.000109	0.979252	1.022185	1.066325	1.242127

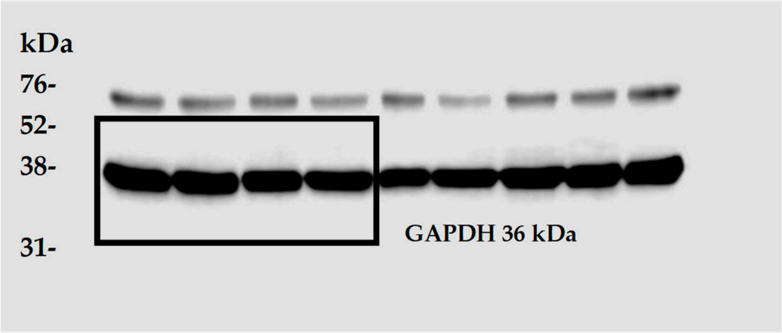

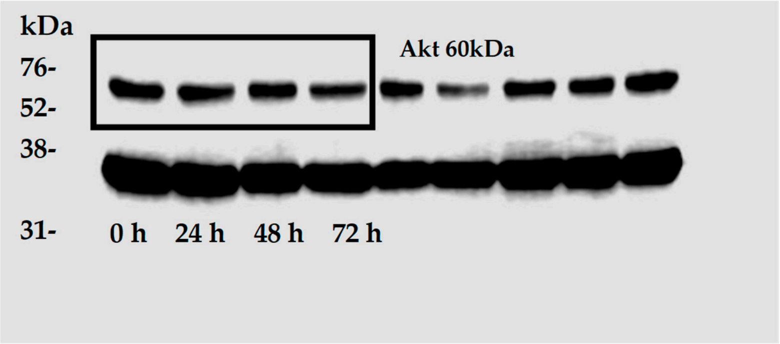

IntDen	S1-M1-80 0 h	S1-M1-80 24 h	S1-M1-80 48 h	S1-M1-80 72 h
Akt	657147	629041	530343	562422
GAPDH	317792	299924	264965	236400
Relative intensity (%)	2.067853	2.097335	2.001559	2.379112

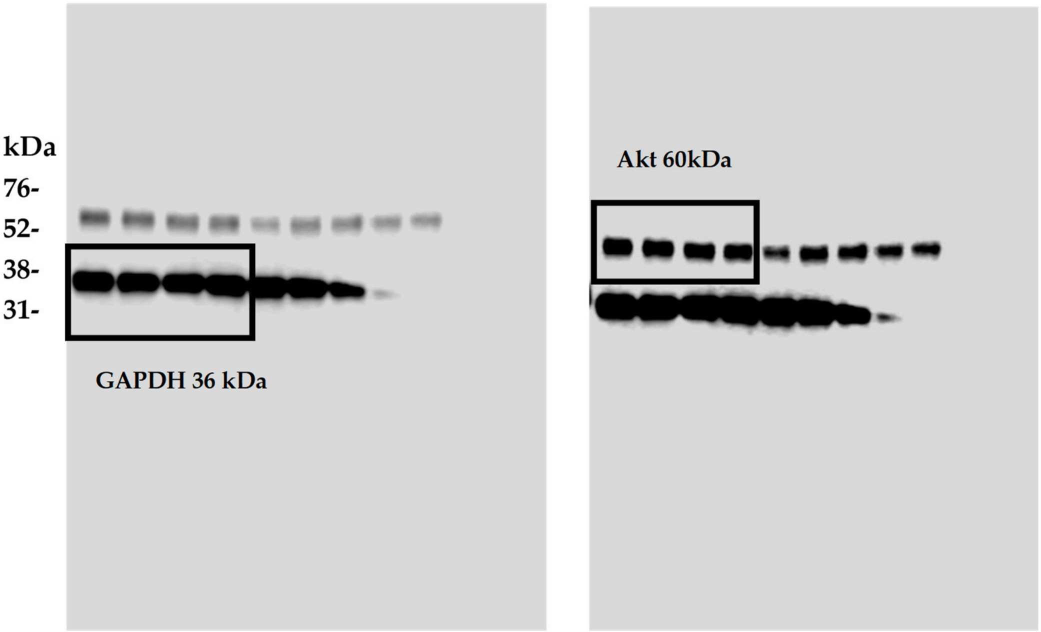

IntDen	SW620/Ad300 0 h	SW620/Ad300 24 h	SW620/Ad300 48 h	SW620/Ad300 72 h
Akt	211552	217560	205772	188850
GAPDH	334241	332130	327950	369428
Relative intensity (%)	0.632933	0.655045	0.627449	0.511196
